# Crystal structure of 3,14-dimethyl-2,6,13,17-tetra­azoniatri­cyclo­[16.4.0.0^7,12^]docosane tetra­chloride tetra­hydrate from synchrotron X-ray data

**DOI:** 10.1107/S2056989018009337

**Published:** 2018-07-06

**Authors:** Dohyun Moon, Jong-Ha Choi

**Affiliations:** a Pohang Accelerator Laboratory, POSTECH, Pohang 37673, Republic of Korea; b Department of Chemistry, Andong National University, Andong 36729, Republic of Korea

**Keywords:** crystal structure, protonated macrocycle, tetra­chloride, tetra­hydrate, hydrogen bonding, synchrotron radiation

## Abstract

The crystal structure of the title salt, C_20_H_44_N_4_^4+^·4Cl^−^·4H_2_O, has been determined using synchrotron radiation at 220 K. The structure determination reveals that protonation has occurred at all four amine N atoms. The asymmetric unit contains one half-cation (completed by crystallographic inversion symmetry), two chloride anions and two water mol­ecules. There are two mol­ecules in the unit cell. The Cl^−^ anions and hydrate mol­ecules are involved in hydrogen bonding. The crystal structure is stabilized by inter­molecular hydrogen bonds involving the macrocycle N—H groups and water O—H groups as donors and the O atoms of the water mol­ecules and the Cl^−^ anions as acceptors, giving rise to a three-dimensional network.

## Abstract 2

In this hydrated 2,6,13,17-tetra­azoniatri­cyclo­[16.4.0.0^7,12^]docosane tetra­chloride salt, the cation lies about an inversion center. In the crystal, N—H⋯Cl, O–H⋯Cl and N—H⋯O hydrogen bonds connect the anions, cations and water mol­ecules, forming a three-dimensional network.

## Chemical context   

The macrocycle 3,14-dimethyl-2,6,13,17-tetra­aza­tri­cyclo­(16.4.0.0^7,12^)docosane (C_20_H_40_N_4_, *L*) is a strongly basic amine capable of forming the [C_20_H_42_N_4_]^2+^ dication or the [C_20_H_44_N_4_]^4+^ tetra­cation in which all of the N—H bonds are generally available for hydrogen-bond formation. These di- or tetra­ammonium cations may be suitable for the removal of toxic heavy metal ions from water. The macrocycle *L* contains a cyclam backbone with two cyclo­hexa­ne subunits. Methyl groups are attached to the 3 and 14 carbon atoms of the propyl chains that bridge opposite pairs of N atoms in the structure. Previously, we have reported the crystal structures of [Cu(*L*)](NO_3_)_2_·3H_2_O, [Cu(*L*)](NO_3_)_2_, [Cu(*L*)](ClO_4_)_2_ and [Cu(*L*)(H_2_O)_2_](BF_4_)_2_·2H_2_O together with [Zn(*L*)(OCOCH_3_)_2_]. In these structures, the copper(II) or zinc(II) cations have tetra­gonally distorted octa­hedral environments with the four N atoms of the macrocyclic ligand in equatorial positions and O atoms of counter-anions, water mol­ecules or acetato ligands in axial positions (Choi *et al.*, 2006 ▸, 2007 ▸, 2012*a* ▸,*b* ▸; Ross *et al.*, 2012 ▸). In these Cu^II^ and Zn^II^ complexes, the macrocyclic ligands adopt their most stable *trans*-III configurations. The crystal structures of the di-cations C_20_H_40_N_4_·2C_11_H_10_O (Choi *et al.*, 2012*c* ▸) and [C_20_H_42_N_4_](SO_4_)·2MeOH (White *et al.*, 2015 ▸) have also been reported. As part of our research program in this area, we report here the preparation of the new tetra-cationic compound, [C_20_H_44_N_4_]Cl_4_·4H_2_O, (I), as the hydrated chloride salt and its structural characterization by synchrotron single-crystal X-ray diffraction.

## Structural commentary   

**Figure 1:**
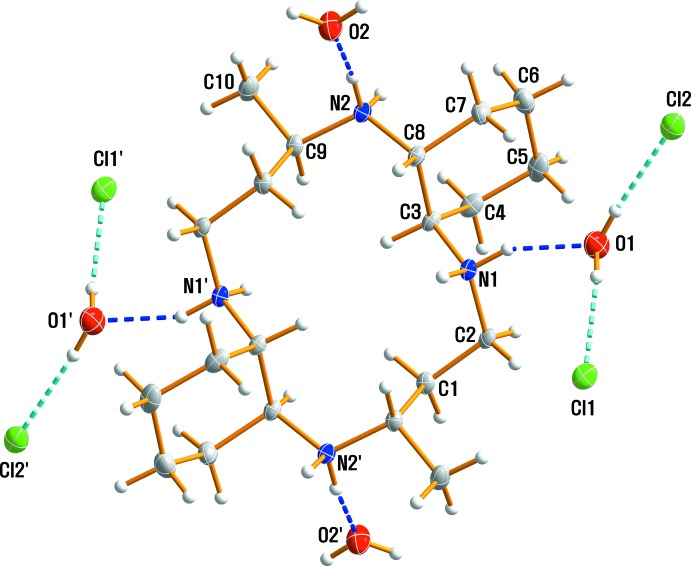
The mol­ecular structure of compound (I), drawn with displacement ellipsoids at the 50% probability level. Dashed lines represent hydrogen-bonding inter­actions and primed atoms are related by the symmetry code (1 − *x*, 1 − *y*, 1 − *z*).

The title compound contains a positively charged macrocyclic cation, 4Cl^−^ anions and four solvent water mol­ecules and was characterized during studies of the macrocyclic ligand and its copper(II) complexes. An ellipsoid plot of the mol­ecular components in (I) with the atom-numbering scheme is shown in Fig. 1 ▸. The asymmetric unit consists of one half of the macrocycle, which lies about a center of inversion, two chloride anions and two solvent water mol­ecules. The four N atoms are coplanar, and the two methyl substituents are *anti* with respect to the macrocyclic plane as a result of the mol­ecular inversion symmetry. The six-membered cyclo­hexane ring is in a stable chair conformation. Within the centrosymmetric tetra-protonated amine unit [C_20_H_44_N_4_]^4+^, the C—C and N—C bond lengths vary from 1.522 (2) to 1.542 (2) Å and from 1.506 (2) to 1.522 (2) Å, respectively. The ranges of N—C—C and C—N—C angles are 106.85 (10) to 114.32 (11)° and 116.70 (10) to 118.89 (10)°, respectively. The bond lengths and angles within the [C_20_H_44_N_4_]^4+^ tetra-cation are comparable to those found in the free ligand or the di-cation in C_20_H_40_N_4_·2C_11_H_10_O (Choi *et al.*, 2012*c* ▸), [C_20_H_42_N_4_](SO_4_)·2MeOH (White *et al.*, 2015 ▸) and [C_20_H_42_N_4_][Fe{HB(pz)_3_}(CN)_3_]_2_·2H_2_O·2MeOH (Kim *et al.*, 2004 ▸).
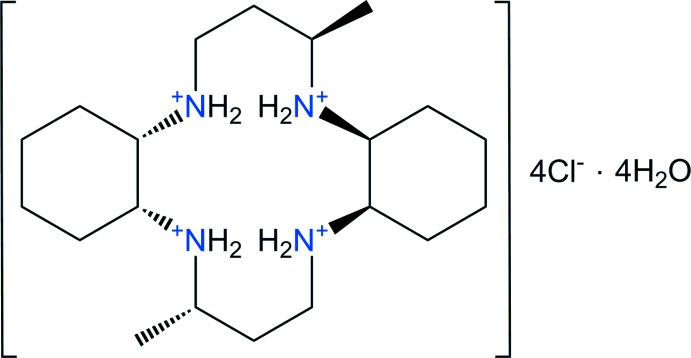


## Supra­molecular features   

**Figure 2:**
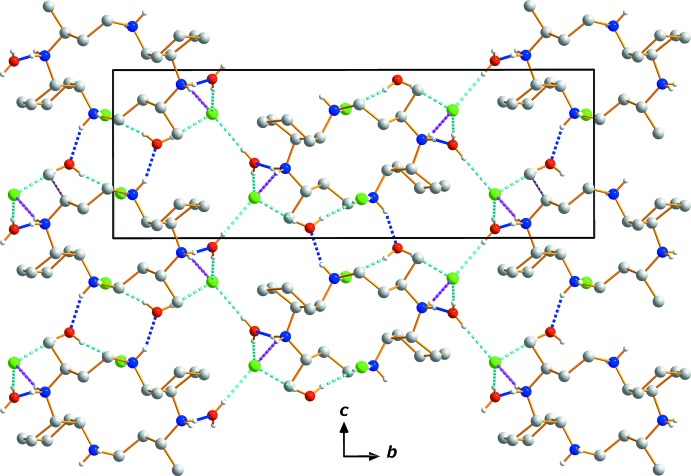
The crystal packing in compound (I), viewed perpendicular to the *bc* plane. Dashed lines represent N—H⋯O (blue), N—H⋯Cl (pink) and O—H⋯Cl (cyan) hydrogen-bonding inter­actions, respectively. C-bound H atoms have been omitted.

Extensive O—H⋯Cl, N—H⋯Cl and N—H⋯O hydrogen-bonding inter­actions occur in the crystal structure (Table 1 ▸). All of the Cl^−^ anions and the O atoms of the water mol­ecules serve as hydrogen-bond acceptors. O—H⋯Cl hydrogen bonds link the water mol­ecules to the neighboring Cl^−^ anions, while N—H⋯Cl and N—H⋯O hydrogen bonds inter­connect the [C_20_H_44_N_4_]^4+^ cations with both anions and water mol­ecules (Figs. 1 ▸ and 2 ▸). The hydrogen atoms on N1 and N2 both form bifurcated hydrogen bonds with O and Cl atoms. The extensive array of these contacts generates a three-dimensional network structure (Fig. 2 ▸), and these hydrogen-bonding inter­actions help to stabilize the crystal structure.

## Database survey   

A search of the Cambridge Structural Database (Version 5.38, May 2017 with three updates; Groom *et al.*, 2016 ▸) gave just three hits for compounds containing the macrocycles [C_20_H_44_N_4_]^4+^, [C_20_H_42_N_4_]^2+^ or (C_20_H_40_N_4_). The crystal structures of C_20_H_40_N_4_·2C_11_H_10_O (Choi *et al.*, 2012*c* ▸), [C_20_H_42_N_4_](SO_4_)·2MeOH (White *et al.*, 2015 ▸) and [C_20_H_42_N_4_][Fe{HB(pz)_3_}(CN)_3_]_2_·2H_2_O·2MeOH (Kim *et al.*, 2004 ▸) were reported previously. However, to our knowledge no crystal structure of any compound with [C_20_H_44_N_4_]^4+^ has been reported.

## Synthesis and crystallization   

Commercially available *trans*-1,2-cyclo­hexa­nedi­amine and methyl vinyl ketone (Sigma–Aldrich) were used as provided. All chemicals were reagent grade and used without further purification. As a starting material, the macrocycle 3,14-dimethyl-2,6,13,17-tetra­aza­tri­cyclo­(16.4.0.0^7,12^)docosane was prepared according to a published procedure (Kang *et al.*, 1991 ▸). A solution of the macrocyclic ligand (0.084 g, 0.25 mmol) in water (10 mL) was added dropwise to a stirred solution of CuCl_2_·2H_2_O (0.085 g, 0.5 mmol) in water (15 mL). The solution was heated for 1 h at 338 K. After cooling to 298 K, the pH was adjusted to 3.0 with 1.0 *M* HCl. The solution was filtered and left at room temperature. Colourless crystals suitable for X-ray analysis were obtained unexpectedly from the solution over a period of a few days.

## Refinement   

Crystal data, data collection and structure refinement details are summarized in Table 2 ▸. All C and N-bound H atoms in the complex were placed in geometrically idealized positions and constrained to ride on their parent atoms, with C—H distances of 0.97–0.99 Å, an N—H distance of 0.9 Å and with *U*_iso_(H) values of 1.2*U*_eq_(C, N) and 1.5*U*_eq_(C-meth­yl). O-bound H atoms of the water mol­ecules were located in a difference-Fourier map, and the O—H distances and the H—O—H angles were restrained using DFIX and DANG constraints (0.84 and 1.36 Å).

## Supplementary Material

Crystal structure: contains datablock(s) I. DOI: 10.1107/S2056989018009337/sj5559sup1.cif

Structure factors: contains datablock(s) I. DOI: 10.1107/S2056989018009337/sj5559Isup2.hkl

Supporting information file. DOI: 10.1107/S2056989018009337/sj5559Isup3.cml

CCDC reference: 1852146

Additional supporting information:  crystallographic information; 3D view; checkCIF report

## Figures and Tables

**Table 1:** Hydrogen-bond geometry (Å, °)

*D*—H⋯*A*	*D*—H	H⋯*A*	*D*⋯*A*	*D*—H⋯*A*
O1—H1*O*1⋯Cl1	0.84 (1)	2.26 (1)	3.0837 (13)	167 (2)
O1—H2*O*1⋯Cl2	0.84 (1)	2.30 (1)	3.1329 (13)	173 (2)
O2—H1*O*2⋯Cl2^i^	0.83 (1)	2.32 (1)	3.1403 (14)	172 (2)
O2—H2*O*2⋯Cl2^ii^	0.83 (1)	2.35 (1)	3.1839 (16)	174 (2)
N1—H1*AN*⋯Cl1^iii^	0.90	2.20	3.0939 (12)	171
N1—H1*B*⋯O1	0.90	1.90	2.7484 (17)	156
N2—H2*AN*⋯Cl2^iv^	0.90	2.41	3.2819 (13)	164
N2—H2*B*⋯O2	0.90	1.85	2.7245 (16)	163

Symmetry codes: (i) 
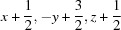
; (ii) 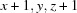; (iii) 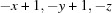; (iv) 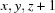.

**Table 2:** Experimental details

Crystal data	
Chemical formula	C_20_H_44_N_4_^4+^·4Cl^−^·4H_2_O
*M*_r_	554.45
Crystal system, space group	Monoclinic, *P*2_1_/*n*
Temperature (K)	220
*a*, *b*, *c* (Å)	7.5450 (15), 23.190 (5), 8.3370 (17)
β (°)	103.32 (3)
*V* (Å^3^)	1419.5 (5)
*Z*	2
Radiation type	Synchrotron, λ = 0.630 Å
μ (mm^−1^)	0.32
Crystal size (mm)	0.08 × 0.06 × 0.06

Data collection	
Diffractometer	Rayonix MX225HS CCD area detector
Absorption correction	Empirical (using intensity measurements) (*HKL3000sm *SCALEPACK**; Otwinowski & Minor, 1997 ▸)
*T*_min_, *T*_max_	0.919, 1.000
No. of measured, independent and observed [*I* > 2σ(*I*)] reflections	13816, 3797, 3504
*R*_int_	0.061
(sin θ/λ)_max_ (Å^−1^)	0.683

Refinement	
*R*[*F* ^2^ > 2σ(*F* ^2^)], *wR*(*F* ^2^), *S*	0.046, 0.141, 1.14
No. of reflections	3797
No. of parameters	158
No. of restraints	6
H-atom treatment	H atoms treated by a mixture of independent and constrained refinement
Δρ_max_, Δρ_min_ (e Å^−3^)	0.80, −0.33

Computer programs: *PAL BL2D-SMDC* (Shin *et al.*, 2016 ▸), *HKL3000sm* (Otwinowski & Minor, 1997 ▸), *SHELXT2015* (Sheldrick, 2015*a* ▸), *SHELXL2015* (Sheldrick, 2015*b* ▸), *DIAMOND* (Putz & Brandenburg, 2014 ▸) and *publCIF* (Westrip, 2010 ▸).

## supplementary crystallographic information

### Crystal data

**Table:**

C_20_H_44_N_4_^4+^·4Cl^−^·4H_2_O	*F*(000) = 600
*M**_r_* = 554.45	*D*_x_ = 1.297 Mg m^−^^3^
Monoclinic, *P*2_1_/*n*	Synchrotron radiation, λ = 0.630 Å
*a* = 7.5450 (15) Å	Cell parameters from 44298 reflections
*b* = 23.190 (5) Å	θ = 0.4–33.6°
*c* = 8.3370 (17) Å	µ = 0.32 mm^−^^1^
β = 103.32 (3)°	*T* = 220 K
*V* = 1419.5 (5) Å^3^	Block, colourless
*Z* = 2	0.08 × 0.06 × 0.06 mm

### Data collection

**Table:**

Rayonix MX225HS CCD area detector diffractometer	3504 reflections with *I* > 2σ(*I*)
Radiation source: PLSII 2D bending magnet	*R*_int_ = 0.061
ω scans	θ_max_ = 25.5°, θ_min_ = 1.6°
Absorption correction: empirical (using intensity measurements) (*HKL3000sm SCALEPACK*; Otwinowski & Minor, 1997)	*h* = −10→10
*T*_min_ = 0.919, *T*_max_ = 1.000	*k* = −31→31
13816 measured reflections	*l* = −11→11
3797 independent reflections	

### Refinement

**Table:**

Refinement on *F*^2^	6 restraints
Least-squares matrix: full	Hydrogen site location: mixed
*R*[*F*^2^ > 2σ(*F*^2^)] = 0.046	H atoms treated by a mixture of independent and constrained refinement
*wR*(*F*^2^) = 0.141	*w* = 1/[σ^2^(*F*_o_^2^) + (0.0863*P*)^2^ + 0.2986*P*] where *P* = (*F*_o_^2^ + 2*F*_c_^2^)/3
*S* = 1.14	(Δ/σ)_max_ = 0.001
3797 reflections	Δρ_max_ = 0.80 e Å^−^^3^
158 parameters	Δρ_min_ = −0.33 e Å^−^^3^

### Special details

**Table:**

Geometry. All esds (except the esd in the dihedral angle between two l.s. planes) are estimated using the full covariance matrix. The cell esds are taken into account individually in the estimation of esds in distances, angles and torsion angles; correlations between esds in cell parameters are only used when they are defined by crystal symmetry. An approximate (isotropic) treatment of cell esds is used for estimating esds involving l.s. planes.

### Fractional atomic coordinates and isotropic or equivalent isotropic displacement parameters (Å^2^)

**Table:**

	*x*	*y*	*z*	*U*_iso_*/*U*_eq_	
Cl1	0.28649 (4)	0.48361 (2)	−0.23231 (4)	0.02152 (13)	
Cl2	0.23041 (5)	0.70611 (2)	−0.24081 (5)	0.02724 (13)	
O1	0.17644 (18)	0.59115 (5)	−0.06048 (14)	0.0292 (3)	
H1O1	0.211 (3)	0.5659 (7)	−0.118 (2)	0.044*	
H2O1	0.190 (3)	0.6234 (5)	−0.101 (3)	0.044*	
O2	0.81871 (15)	0.70598 (5)	0.55335 (16)	0.0285 (3)	
H1O2	0.807 (3)	0.7293 (8)	0.476 (2)	0.043*	
H2O2	0.9243 (18)	0.7082 (9)	0.612 (2)	0.043*	
N1	0.31217 (15)	0.54018 (5)	0.24038 (13)	0.0135 (2)	
H1AN	0.424887	0.529030	0.234909	0.016*	
H1B	0.273908	0.566098	0.159468	0.016*	
N2	0.52610 (14)	0.64198 (5)	0.58563 (14)	0.0145 (2)	
H2AN	0.431936	0.660340	0.612559	0.017*	
H2B	0.612982	0.668566	0.585565	0.017*	
C1	0.24830 (16)	0.43768 (5)	0.32273 (16)	0.0151 (2)	
H1A	0.142456	0.413129	0.322216	0.018*	
H1AB	0.292168	0.452568	0.434890	0.018*	
C2	0.18858 (17)	0.48834 (5)	0.20562 (17)	0.0151 (3)	
H2A	0.182324	0.475365	0.092579	0.018*	
H2AB	0.065641	0.500024	0.212226	0.018*	
C3	0.32531 (16)	0.57005 (5)	0.40305 (15)	0.0139 (2)	
H3	0.374735	0.542524	0.493008	0.017*	
C4	0.13998 (17)	0.59125 (6)	0.42271 (17)	0.0190 (3)	
H4A	0.055377	0.558611	0.409763	0.023*	
H4AB	0.152601	0.606815	0.534063	0.023*	
C5	0.06125 (19)	0.63767 (7)	0.29694 (19)	0.0240 (3)	
H5A	−0.055674	0.650884	0.315737	0.029*	
H5AB	0.039283	0.621385	0.185629	0.029*	
C6	0.1913 (2)	0.68874 (7)	0.31020 (19)	0.0257 (3)	
H6A	0.199462	0.708191	0.416010	0.031*	
H6AB	0.142341	0.716389	0.222244	0.031*	
C7	0.38260 (19)	0.67021 (6)	0.29703 (18)	0.0205 (3)	
H7A	0.378106	0.658961	0.182857	0.025*	
H7AB	0.464730	0.703370	0.323138	0.025*	
C8	0.46174 (16)	0.62004 (5)	0.41108 (15)	0.0141 (2)	
H8	0.569077	0.605031	0.374969	0.017*	
C9	0.60141 (16)	0.59907 (5)	0.72246 (16)	0.0144 (2)	
H9	0.501674	0.573469	0.737922	0.017*	
C10	0.6708 (2)	0.63373 (6)	0.87937 (17)	0.0233 (3)	
H10A	0.706638	0.607639	0.972125	0.035*	
H10B	0.574944	0.659033	0.897628	0.035*	
H10C	0.774932	0.656615	0.868372	0.035*	

### Atomic displacement parameters (Å^2^)

**Table:**

	*U*^11^	*U*^22^	*U*^33^	*U*^12^	*U*^13^	*U*^23^
Cl1	0.01190 (19)	0.0294 (2)	0.0260 (2)	−0.00148 (11)	0.01001 (14)	−0.00336 (13)
Cl2	0.0223 (2)	0.0314 (2)	0.0275 (2)	0.00606 (13)	0.00466 (16)	−0.00617 (14)
O1	0.0377 (6)	0.0317 (6)	0.0187 (5)	0.0038 (5)	0.0078 (5)	−0.0001 (4)
O2	0.0175 (5)	0.0358 (6)	0.0316 (6)	−0.0066 (4)	0.0043 (5)	0.0089 (5)
N1	0.0089 (4)	0.0188 (5)	0.0133 (5)	0.0003 (4)	0.0036 (4)	−0.0002 (4)
N2	0.0105 (5)	0.0176 (5)	0.0151 (5)	−0.0007 (4)	0.0024 (4)	−0.0005 (4)
C1	0.0102 (5)	0.0195 (5)	0.0163 (6)	0.0012 (4)	0.0045 (4)	0.0007 (5)
C2	0.0093 (5)	0.0183 (5)	0.0162 (6)	−0.0006 (4)	−0.0001 (4)	−0.0021 (5)
C3	0.0106 (5)	0.0192 (5)	0.0124 (5)	−0.0028 (4)	0.0036 (4)	−0.0016 (4)
C4	0.0121 (6)	0.0275 (6)	0.0198 (6)	−0.0038 (5)	0.0083 (5)	−0.0062 (5)
C5	0.0130 (6)	0.0349 (7)	0.0236 (7)	0.0061 (5)	0.0035 (5)	−0.0039 (6)
C6	0.0254 (7)	0.0249 (6)	0.0250 (7)	0.0071 (6)	0.0024 (6)	0.0034 (6)
C7	0.0188 (6)	0.0223 (6)	0.0189 (6)	−0.0018 (5)	0.0012 (5)	0.0054 (5)
C8	0.0104 (5)	0.0192 (5)	0.0127 (5)	−0.0026 (4)	0.0024 (4)	−0.0004 (5)
C9	0.0106 (5)	0.0190 (5)	0.0137 (6)	0.0008 (4)	0.0028 (4)	0.0009 (4)
C10	0.0242 (7)	0.0278 (7)	0.0159 (6)	0.0051 (6)	0.0004 (5)	−0.0054 (5)

### Geometric parameters (Å, º)

**Table:**

O1—H1O1	0.837 (9)	C3—H3	0.9900
O1—H2O1	0.837 (9)	C4—C5	1.524 (2)
O2—H1O2	0.833 (9)	C4—H4A	0.9800
O2—H2O2	0.834 (9)	C4—H4AB	0.9800
N1—C3	1.5056 (16)	C5—C6	1.526 (2)
N1—C2	1.5080 (16)	C5—H5A	0.9800
N1—H1AN	0.9000	C5—H5AB	0.9800
N1—H1B	0.9000	C6—C7	1.534 (2)
N2—C8	1.5122 (17)	C6—H6A	0.9800
N2—C9	1.5217 (16)	C6—H6AB	0.9800
N2—H2AN	0.9000	C7—C8	1.5338 (18)
N2—H2B	0.9000	C7—H7A	0.9800
C1—C2	1.5277 (18)	C7—H7AB	0.9800
C1—C9^i^	1.5333 (17)	C8—H8	0.9900
C1—H1A	0.9800	C9—C10	1.5217 (19)
C1—H1AB	0.9800	C9—H9	0.9900
C2—H2A	0.9800	C10—H10A	0.9700
C2—H2AB	0.9800	C10—H10B	0.9700
C3—C4	1.5263 (17)	C10—H10C	0.9700
C3—C8	1.5416 (17)		
			
H1O1—O1—H2O1	108.0 (17)	H4A—C4—H4AB	107.9
H1O2—O2—H2O2	108.9 (18)	C4—C5—C6	110.86 (12)
C3—N1—C2	116.70 (10)	C4—C5—H5A	109.5
C3—N1—H1AN	108.1	C6—C5—H5A	109.5
C2—N1—H1AN	108.1	C4—C5—H5AB	109.5
C3—N1—H1B	108.1	C6—C5—H5AB	109.5
C2—N1—H1B	108.1	H5A—C5—H5AB	108.1
H1AN—N1—H1B	107.3	C5—C6—C7	112.18 (12)
C8—N2—C9	118.89 (10)	C5—C6—H6A	109.2
C8—N2—H2AN	107.6	C7—C6—H6A	109.2
C9—N2—H2AN	107.6	C5—C6—H6AB	109.2
C8—N2—H2B	107.6	C7—C6—H6AB	109.2
C9—N2—H2B	107.6	H6A—C6—H6AB	107.9
H2AN—N2—H2B	107.0	C8—C7—C6	113.98 (11)
C2—C1—C9^i^	113.35 (10)	C8—C7—H7A	108.8
C2—C1—H1A	108.9	C6—C7—H7A	108.8
C9^i^—C1—H1A	108.9	C8—C7—H7AB	108.8
C2—C1—H1AB	108.9	C6—C7—H7AB	108.8
C9^i^—C1—H1AB	108.9	H7A—C7—H7AB	107.7
H1A—C1—H1AB	107.7	N2—C8—C7	109.53 (10)
N1—C2—C1	114.32 (11)	N2—C8—C3	110.97 (10)
N1—C2—H2A	108.7	C7—C8—C3	112.51 (11)
C1—C2—H2A	108.7	N2—C8—H8	107.9
N1—C2—H2AB	108.7	C7—C8—H8	107.9
C1—C2—H2AB	108.7	C3—C8—H8	107.9
H2A—C2—H2AB	107.6	C10—C9—N2	107.10 (11)
N1—C3—C4	111.91 (10)	C10—C9—C1^i^	112.18 (11)
N1—C3—C8	106.85 (10)	N2—C9—C1^i^	110.41 (10)
C4—C3—C8	111.85 (11)	C10—C9—H9	109.0
N1—C3—H3	108.7	N2—C9—H9	109.0
C4—C3—H3	108.7	C1^i^—C9—H9	109.0
C8—C3—H3	108.7	C9—C10—H10A	109.5
C5—C4—C3	112.03 (11)	C9—C10—H10B	109.5
C5—C4—H4A	109.2	H10A—C10—H10B	109.5
C3—C4—H4A	109.2	C9—C10—H10C	109.5
C5—C4—H4AB	109.2	H10A—C10—H10C	109.5
C3—C4—H4AB	109.2	H10B—C10—H10C	109.5
			
C3—N1—C2—C1	−64.11 (14)	C9—N2—C8—C3	52.13 (14)
C9^i^—C1—C2—N1	−80.89 (14)	C6—C7—C8—N2	−76.56 (14)
C2—N1—C3—C4	−58.63 (14)	C6—C7—C8—C3	47.36 (16)
C2—N1—C3—C8	178.63 (10)	N1—C3—C8—N2	−163.70 (10)
N1—C3—C4—C5	−64.60 (14)	C4—C3—C8—N2	73.52 (13)
C8—C3—C4—C5	55.26 (15)	N1—C3—C8—C7	73.18 (13)
C3—C4—C5—C6	−57.49 (15)	C4—C3—C8—C7	−49.60 (15)
C4—C5—C6—C7	54.14 (16)	C8—N2—C9—C10	174.85 (11)
C5—C6—C7—C8	−49.87 (17)	C8—N2—C9—C1^i^	52.43 (14)
C9—N2—C8—C7	176.95 (10)		

Symmetry code: (i) −*x*+1, −*y*+1, −*z*+1.

### Hydrogen-bond geometry (Å, º)

**Table:**

*D*—H···*A*	*D*—H	H···*A*	*D*···*A*	*D*—H···*A*
O1—H1*O*1···Cl1	0.84 (1)	2.26 (1)	3.0837 (13)	167 (2)
O1—H2*O*1···Cl2	0.84 (1)	2.30 (1)	3.1329 (13)	173 (2)
O2—H1*O*2···Cl2^ii^	0.83 (1)	2.32 (1)	3.1403 (14)	172 (2)
O2—H2*O*2···Cl2^iii^	0.83 (1)	2.35 (1)	3.1839 (16)	174 (2)
N1—H1*AN*···Cl1^iv^	0.90	2.20	3.0939 (12)	171
N1—H1*B*···O1	0.90	1.90	2.7484 (17)	156
N2—H2*AN*···Cl2^v^	0.90	2.41	3.2819 (13)	164
N2—H2*B*···O2	0.90	1.85	2.7245 (16)	163

Symmetry codes: (ii) *x*+1/2, −*y*+3/2, *z*+1/2; (iii) *x*+1, *y*, *z*+1; (iv) −*x*+1, −*y*+1, −*z*; (v) *x*, *y*, *z*+1.
